# Seasonality of Rotavirus in South Asia: A Meta-Analysis Approach Assessing Associations with Temperature, Precipitation, and Vegetation Index

**DOI:** 10.1371/journal.pone.0038168

**Published:** 2012-05-31

**Authors:** Jyotsna S. Jagai, Rajiv Sarkar, Denise Castronovo, Deepthi Kattula, Jesse McEntee, Honorine Ward, Gagandeep Kang, Elena N. Naumova

**Affiliations:** 1 National Health and Environmental Effects Research Laboratory, Office of Research and Development, U.S. Environmental Protection Agency, Research Triangle Park, North Carolina, United States of America; 2 Department of Public Health and Community Medicine, Tufts University School of Medicine, Boston, Masschusetts, United States of America; 3 Christian Medical College, Vellore, India; 4 Mapping Sustainability, LLC, Jupiter, Florida, United States of America; 5 The ESRC Centre for Business Relationships, Accountability, Sustainability and Society, Cardiff University, Cardiff, Wales, United Kingdom; 6 Department of Civil and Environmental Engineering, Tufts University School of Engineering, Medford, Massachusetts, United States of America; National Institutes of Health, United States of America

## Abstract

**Background:**

Rotavirus infection causes a significant proportion of diarrhea in infants and young children worldwide leading to dehydration, hospitalization, and in some cases death. Rotavirus infection represents a significant burden of disease in developing countries, such as those in South Asia.

**Methods:**

We conducted a meta-analysis to examine how patterns of rotavirus infection relate to temperature and precipitation in South Asia. Monthly rotavirus data were abstracted from 39 published epidemiological studies and related to monthly aggregated ambient temperature and cumulative precipitation for each study location using linear mixed-effects models. We also considered associations with vegetation index, gathered from remote sensing data. Finally, we assessed whether the relationship varied in tropical climates and humid mid-latitude climates.

**Results:**

Overall, as well as in tropical and humid mid-latitude climates, low temperature and precipitation levels are significant predictors of an increased rate of rotaviral diarrhea. A 1°C decrease in monthly ambient temperature and a decrease of 10 mm in precipitation are associated with 1.3% and 0.3% increase above the annual level in rotavirus infections, respectively. When assessing lagged relationships, temperature and precipitation in the previous month remained significant predictors and the association with temperature was stronger in the tropical climate. The same association was seen for vegetation index; a seasonal decline of 0.1 units results in a 3.8% increase in rate of rotavirus.

**Conclusions:**

In South Asia the highest rate of rotavirus was seen in the colder, drier months. Meteorological characteristics can be used to better focus and target public health prevention programs.

## Introduction

Rotavirus causes a significant proportion of diarrhea in infants and young children worldwide leading to dehydration, hospitalization and in some cases death. It has been estimated that the proportion of hospitalizations for childhood diarrhea due to rotavirus has increased from 22% to 39% from 1986 to 2004 [1] and for 2004 there were an estimated 527,000 rotavirus-related deaths annually worldwide [2]. Rotavirus is a common infection in children worldwide and it is estimated that by age 5 nearly every child will have an episode of rotavirus infection [3], [4]. Children in developing countries, such as those in South Asia, bear the greatest burden of deaths, up to 82%, from rotavirus infection, primarily due to malnutrition or lack of access to rehydration therapy [3]. A recent review on the burden of rotavirus diarrhea estimated that rotavirus is responsible for the death of approximately 122,000–153,000 children annually in India alone [4]. As of 2006, there are two live oral vaccines which have undergone large scale clinical trials and are being introduced globally [5], [6]. These vaccines may have a significant impact in developing countries. Increased understanding of the epidemiology and, in particular, seasonal patterns of rotavirus in these countries is needed to ensure effective vaccine intervention programs [6].

Seasonal fluctuations in infectious diseases, and specifically in rotavirus infection, are a well-known and well documented phenomenon [7], [8]. Rotavirus manifests at low endemic levels with pronounced seasonal outbreaks with considerable geographic variation. The virus is considered a winter disease with most cases observed in the coldest time of the year [7]–[9]. A review of 34 studies conducted worldwide found that rotavirus is more common in the cooler months in the temperate zones, yet the peak of disease can vary from autumn to spring [7]. The strong winter peak was seen primarily in the Americas, though in cities which cover various climates such as Toronto, Ontario, Canada and Houston, Texas, USA [7]. However in tropics (within 10N or 10S of the Equator) the pattern is less defined and autumn/spring peaks are more common [7]. A recent review considered the tropical region specifically and associated monthly disease incidence with meteorological variables for the same month. The analysis found that a 1°C increase in mean temperature resulted in a 10% decrease in rotavirus incidence and a 1 cm increase in mean monthly rainfall was associated with a 1% decrease in rotavirus incidence [8]. A review of 43 studies conducted in Africa found that rotavirus was detected year-round in nearly every country and generally exhibited distinct seasonal peaks during the dry months [10]. Another study of hospitalization for rotavirus which was conducted in three Australian cities found that admissions for rotavirus diarrhea peaked in winter and spring and was lowest in summer [11]. They also found that higher temperature and humidity in the previous week were associated with a decrease in rotavirus diarrheal admissions in all three cities [11].

The core of seasonality in infectious diseases is thought to be related to temporal oscillations in the governing transmission cycles of pathogenic agents and host susceptibility. Seasonal factors operate at many levels, creating temporal changes in human behavior, including sanitation and hygiene practices, and probability of environmental exposures [7], [12]–[16]. Despite growing attention to disease seasonality, a solid theoretical underpinning for seasonal peaks is limited. Better insight into the nature of disease seasonality can be gained from understanding the association with meteorological and environmental drivers. With increasing monitoring of rotavirus worldwide [10], [17], a more detailed documentation of seasonality will soon be available.

In developing countries consistent meteorological monitoring data is limited therefore satellite imagery can be advantageous and used as a proxy for the combined effects of temperature and precipitation. Remote sensing data are primarily used to derive characteristics related to vegetation cover, landscape structure, and water content. The Vegetation Index (VI) is a measure of density of plant growth which is calculated from remote sensing data. Very low values of VI (0.1 and below) correspond to barren areas of rock, sand, or snow. Moderate values represent shrub and grassland (0.2 to 0.3), while high values indicate temperate and tropical rainforests (0.6 to 0.8). Measures of the VI have been used in previous epidemiological studies [18] and has demonstrated predictive properties for onchocerciasis in Ethiopia [19], schistosomiasis in Brazil [20], West Nile Virus in New York City [21], and cryptosporidiosis for specific climates in Africa [22].

The objective of this study is to increase the understanding of seasonality of rotavirus diarrhea and associations with meteorological characteristics in South Asia. We assembled a set of time series representing monthly rate of rotavirus from 39 studies conducted in South Asia and examined the relationships between disease rate and meteorological factors, namely precipitation and ambient temperature. We also consider the association between rate of rotavirus and vegetation index, a remote sensing measure of the combined effects of temperature and precipitation.

## Methods

### Outcome Data

We conducted a meta-analysis of studies published on rotavirus infection from 1966 to 2010 in South Asia, defined as India, Pakistan, Bangladesh, Sri Lanka, Nepal, and Bhutan. Literature was gathered using the Ovid MEDLINE search engine. The keywords used in the search were *rotavirus infections* or *rotavirus*. These keywords were combined with *seasonality* and *seasons*, resulting in 567 citations. The search was further narrowed by adding the keywords *Asia, Asia Central, Asia Western, Asia Southeastern* resulting in a total of 152 citations after duplicate citations were removed. This database of studies was supplemented with references from review articles of rotavirus [7], [8], [16] and with studies from our own archives that included studies which do not appear in Ovid MEDLINE as they are published in South Asian scientific journals. This resulted in a total of 166 studies for evaluation. Studies were evaluated based on the following four criteria:

1. studies must provide observational data on rotavirus in humans who are not immuno-compromised (i.e., HIV/AIDS patients);
2. studies must include at least a full year of data to cover all seasons;
3. studies must provide disease incidence or prevalence data on a daily, weekly, or monthly aggregation;
4. studies must provide data for a single location (i.e., hospital, village, etc).

**Figure 1:**
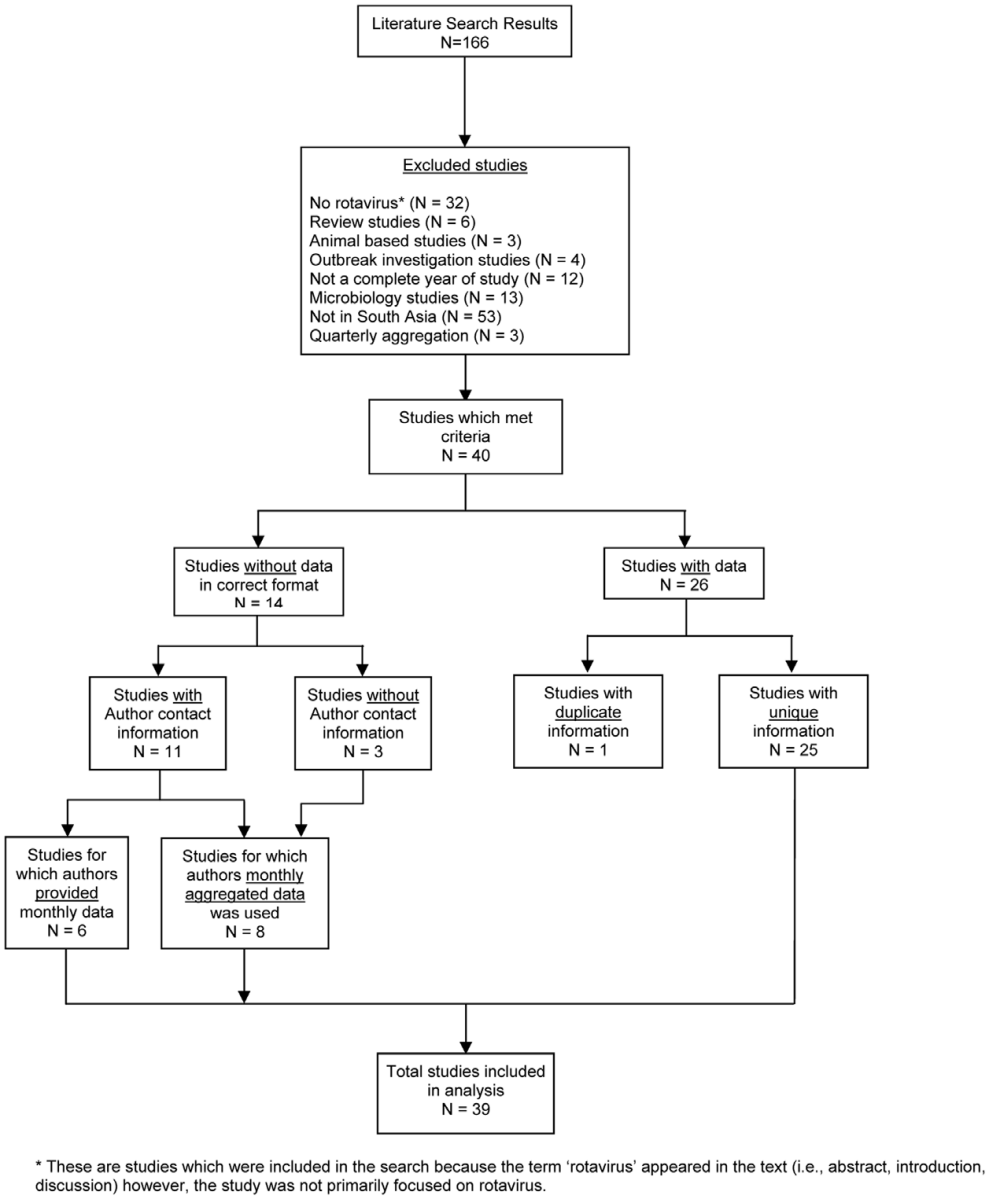
Study selection flow chart. Flow chart of selection of studies utilized in the meta-analysis including inclusion and exclusion criteria.

Studies which did not meet the criteria were excluded (Figure 1). A total of 40 studies met the study criteria of which 26 studies presented data in the published journal article (2 presented data on the same cohort; therefore the duplicate study was not included). We contacted the authors of 11 studies; 6 were able to provide data in the requested format. The remaining 8 studies provided monthly data aggregated over the entire study period. The final analysis comprised data from 39 studies. Data on rotavirus were aggregated on a monthly basis producing 47 time series, reflecting discrete time periods and locations (Table 1). Four studies provided data for more than one location or period of time.

**Table 1:** Study reference, location of study, subjects' age, study setting, climate, latitude, longitude, study start and end date period of data for each times series used in analysis.

Time Series	Study Reference	Location (City, Country)	Subjects' Age (Years)	Study Setting	# of Specimen Tested	Rotavirus Infection (%)	Climate *	Latitude	Longitude	Study Start Date	Study End Date	Study Duration (months)**	Remote Sensing Data ***
1	[44]	Chandigarh, India	<5 y	Hospital	694	111 (15.9)	C	30°44′N	76°47′E	3/1/1982	12/31/1985	46	GIMMS
2	[45]	Chandigarh, India	<3 y	Community	1024	120 (11.7)	C	30°44′N	76°47′E	12/1/1984	5/31/1987	(30)	GIMMS
3	[46]	Chandigarh, India	<5 yr	Community	218	25 (11.5)	C	30°44′N	76°47′E	10/1/1988	2/28/1991	29	GIMMS
4	[47]	New Delhi, India	0–5 y	Hospital	104	990 (10.5)	C	28°36′N	77°12′E	5/1/1988	5/31/1990	25	GIMMS
5	[48]	New Delhi, India	<3 y	Hospital	400	23 (5.75)	C	28°36′N	77°12′E	6/1/1989	5/31/1990	12	GIMMS
6	[49]	New Delhi, India	<5 y	Community	584	137 (23.5)	C	28°36′N	77°12′E	8/1/2000	7/31/2001	12	MODIS
7	[50]	New Delhi, India	<18 y	Hospital	1524	465 (30.5)	C	28°36′N	77°12′E	8/1/2000	7/31/2007	84	MODIS
8	[51]	New Delhi, India	<2 y	Hospital	862	318 (36.9)	C	28°36′N	77°12′E	2/1/2005	3/31/2007	26	MODIS
9	[52]	New Delhi, India	0–5 y	Hospital	633	232 (36.7)	C	28°36′N	77°12′E	11/1/2005	11/30/2007	25	MODIS
10	[53]	Kathmandu,Nepal	0–5 y	Hospital	1139	379 (33.2)	C	27°42′N	85°12′E	11/1/2005	10/31/2007	24	MODIS
11	[54]	Dibrugarh, India	<5 y	Hospital	202	47 (23.3)	C	27°30′N	95°0′E	4/1/1999	3/31/2000	12	GIMMS
12	[55]	Lucknow, India	<3 y	Hospital	412	79 (19.2)	C	26°55′N	80°59′E	9/1/2004	4/31/2008	(44)	MODIS
13	[56]	Varanasi, India	<5 y	Community	376	67 (17.7)	C	25°19′N	83°0′E	8/1/1988	7/31/1989	12	GIMMS
14	[57]	Karachi, Pakistan	<5 y	Hospital	818	112 (13.7)	B	24°52′N	67°2′E	1/1/1990	12/31/1997	(96)	GIMMS
15	[58]	Karachi, Pakistan	<5 y	Community	575	97 (16.8)	B	24°52′N	67°2′E	6/1/2005	6/30/2007	25	MODIS
16	[59]	Dhaka, Bangladesh	All ages	Hospital	3280	623 (19.0)	A	23°45′N	90°15′E	12/1/1979	11/30/1980	12	N/A
17	[60]	Dhaka, Bangladesh	All ages	Hospital	1258	217 (17.2)	A	23°45′N	90°15′E	1/1/1983	12/31/1984	24	GIMMS
18	[61]	Dhaka, Bangladesh	<5 y	Hospital	111	35 (32.0)	A	23°45′N	90°15′E	1/1/1989	12/31/1989	12	GIMMS
19	[62]	Dhaka, Bangladesh	All ages	Hospital	4335	756 (17.4)	A	23°45′N	90°15′E	6/1/1989	7/31/1990	14	GIMMS
20	[63]	Dhaka, Bangladesh	<5 y	Hospital	7709	1561 (20.2)	A	23°45′N	90°15′E	1/1/1990	12/31/1993	48	GIMMS
21	[64]	Dhaka, Bangladesh	<5 y	Hospital	28899	9066 (31.4)	A	23°45′N	90°15′E	1/1/1993	12/31/2004	(144)	MODIS
22	[65]	Dhaka, Bangladesh	All ages	Hospital	10739	2706 (25.2)	A	23°45′N	90°15′E	1/1/2001	5/31/2006	65	MODIS
23	[66]	Dhaka, Bangladesh	<2 y	Community	1181	121 (10.2)	A	23°45′N	90°15′E	4/1/2002	10/30/2004	(31)	MODIS
24	[67]	Dhaka, Bangladesh	<5 y	Hospital	656	259 (39.5)	A	23°45′N	90°15′E	7/1/2005	6/31/2006	13	MODIS
25	[68]	Matlab, Bangladesh	0–4 y	Community	920	35 (3.8)	A	23°21′N	90°45′E	4/1/1978	3/31/1979	12	N/A
26	[69]	Matlab, Bangladesh	All ages	Hospital	5811	898 (15.5)	A	23°21′N	90°45′E	6/1/1987	5/31/1989	24	GIMMS
27	[62]	Matlab, Bangladesh	All ages	Hospital	570	135 (23.7)	A	23°21′N	90°45′E	6/1/1989	7/31/1990	14	GIMMS
28	[70]	Matlab, Bangladesh	<5 y	Hospital	4519	1479 (32.7)	A	23°21′N	90°45′E	1/1/2000	12/31/2006	(84)	MODIS
29	[65]	Matlab, Bangladesh	All ages	Hospital	8300	1938 (23.3)	A	23°21′N	90°45′E	1/1/2001	5/31/2006	65	MODIS
30	[71]	Kolkata, India	0–12 y	Hospital	245	55 (22.4)	A	22°31′N	88°25′E	7/1/1979	6/30/1981	24	N/A
31	[52]	Kolkata, India	0–5 y	Hospital	394	179 (45.5)	A	22°31′N	88°25′E	11/1/2005	11/30/2007	25	MODIS
32	[72]	Kolkata, India	All ages	Hospital	2519	493 (19.6)	A	22°31′N	88°25′E	11/1/2007	10/31/2009	24	MODIS
33	[52]	Mumbai, India	0–5 y	Hospital	745	263 (35.3)	A	18°54′N	72°49′E	11/1/2005	11/30/2007	25	MODIS
34	[73]	Pune, India	All ages	Hospital	945	266 (28.1)	A	18°45′N	73°45′E	7/1/1992	6/30/1996	48	GIMMS
35	[74]	Pune, India	>10 y	Hospital	1338	75 (5.6)	A	18°45′N	73°45′E	1/1/1993	12/31/1996	(48)	GIMMS
36	[74]	Pune, India	>10 y	Hospital	253	43 (17.0)	A	18°45′N	73°45′E	1/1/2004	12/31/2007	(48)	MODIS
37	[52]	Pune, India	0–5 y	Hospital	684	256 (37.4)	A	18°45′N	73°45′E	11/1/2005	11/30/2007	25	MODIS
38	[75]	Yangon, Myanmar	<5 y	Hospital	1736	923 (53.2)	A	16°46′N	96°10′E	1/1/2002	12/31/2003	24	MODIS
39	[76]	Chennai, India	<3 yr	Hospital	245	51 (20.8)	A	13°4′N	80°15′E	12/1/1997	3/31/1999	16	GIMMS
40	[77]	Bangalore, India	<5 y	Hospital	379	62 (16.3)	A	12°58′N	77°34′E	1/1/1983	12/31/1983	12	GIMMS
41	[78]	Vellore, India	<3 y	Hospital	916	163 (17.8)	A	12°55′N	79°11′E	8/1/1983	7/31/1985	24	GIMMS
42	[79]	Vellore, India	<5 yr	Hospital	1495	176 (11.8)	A	12°55′N	79°11′E	1/1/2002	12/31/2003	24	MODIS
43	[52]	Vellore, India	0–5 y	Hospital	718	259 (36.1)	A	12°55′N	79°11′E	11/1/2005	11/30/2007	25	MODIS
44	[80]	Calicut, India	<5 yr	Hospital	365	259 (70.9)	A	11°15′N	75°46′E	9/1/1976	2/28/1978	18	N/A
45	[52]	Trichy, India	0–5 y	Hospital	406	216 (53.2)	A	10°48′N	78°41′E	11/1/2005	11/30/2007	25	MODIS
46	[81]	Colombo, Sri Lanka	<10 y	Hospital	587	118 (20.1)	A	10°48′N	78°41′E	4/1/2005	10/31/2006	19	MODIS
47	[82]	Colombo, Sri Lanka	0–5 y	Hospital	1806	428 (23.7)	A	6°54′N	79°52′E	11/1/2005	11/30/2007	(25)	MODIS

* Climate categories: A - moist tropical climates, B - arid and semiarid climates, C - humid mid-latitude areas, D - colder temperate areas.

** The duration in parenthesis indicates that the monthly aggregated average values were used for this study.

*** Remote sensing data source: GIMMS – Global Inventory Monitoring and Mapping Studies, MODIS – NASA's Moderate-resolution Imaging Spectroradiometer.

### Exposure Data: Temperature and Precipitation

**Figure 2:**
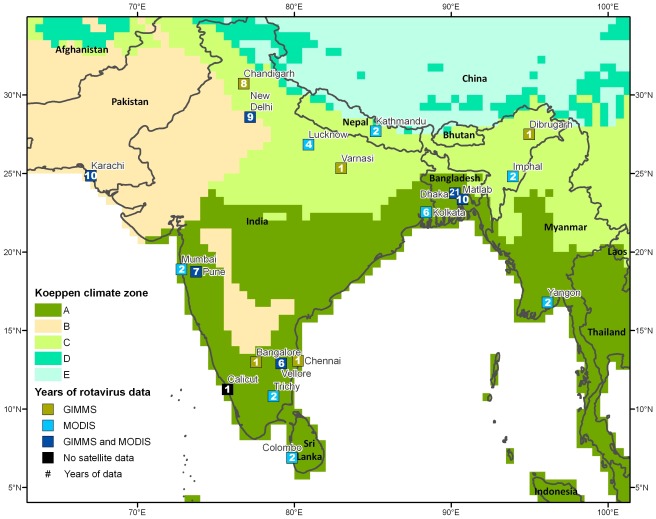
Map of study locations on Köppen Climate. Study locations mapped on Köppen Climate Classification system (Climate categories: A - moist tropical climates, B - arid and semiarid climates, C - humid mid-latitude areas, D - colder temperate areas) with indication of source of remote sensing data (GIMMS – Global Inventory Monitoring and Mapping Studies, MODIS – NASA's Moderate-resolution Imaging Spectroradiometer) and number of years of data available.

Based on the latitude and longitude of the study location for each times series, we supplemented monthly rotavirus data with time specific monthly mean ambient temperature and monthly cumulative precipitation, obtained from the National Climatic Data Center databases (792 of 1046 months = 75.7%). When meteorological data specific to the time of the study were unavailable (254 of 1046 months = 24.3%), normals for monthly average temperature and cumulative precipitation were gathered from Global Historical Climatology Network. Each study location was classified based on the Köppen Climate Classification [23]. Using the study locations' latitude and longitude information each study location was superimposed on a map of Köppen Climate Classification and using a GIS spatial overlay each location was classified into a specific climate category. For the purposes of analysis we used only the four major classifications; moist tropical climates (Climate A), arid and semiarid climates (Climate B), humid mid-latitude areas (Climate C) and colder temperate areas (Climate D) (Figure 2).

### Exposure Data: Vegetation Index

Vegetation index (VI) data were obtained from two sources: the Global Inventory Monitoring and Mapping Studies (GIMMS) data for locations with study dates from 1981 to 2000 and the NASA's Moderate-resolution Imaging Spectroradiometer (MODIS) Terra satellite for locations with study dates from 2000 to 2007. GIMMS data are hosted by the Global Land Cover Facility of the University of Maryland [24], and are derived from Advanced Very High Resolution Radiometer (AVHRR) sensors on board the National Oceanic and Atmospheric Administration's (NOAA) satellite series: NOAA-7, NOAA-9, NOAA-11, NOAA-14 and NOAA-16. GIMMS processing aims to create a consistent time series of the Normalized Difference Vegetation Index (NDVI) from the AVHRR satellite series by correcting for differences in solar illumination angles and sensor view angles over the period of record, caused by satellite overpass time drift. GIMMS processing also reduces NDVI variations that arise from sensor band calibration, volcanic aerosols, cloud cover and water vapor. We acquired GIMMS data on a bimonthly basis as a normalized difference vegetation index (NDVI) composite product with 1 km spatial resolution [24]. MODIS Terra data are hosted by NASA's Warehouse Inventory Search Tool (WIST) and are available from 2000 to present at temporal resolutions of 16 days and 1 month and spatial resolutions of 250 m, 500 m, 1 km and 0.25 degrees. From the MODIS Terra satellite, we acquired monthly composite Enhanced Vegetation Index (EVI) data at 1 km resolution. We chose 1 km spatial resolution to remain consistent with the GIMMS data. Although MODIS data are available on a bimonthly basis, we acquired monthly composite data to help remove errors caused by clouds, sun glare, and the satellite instrument itself [25] and to match the monthly rotavirus time series. The MODIS sensor has many advantages over the AVHRR satellite series for calculating vegetation indices. MODIS offers a higher spatial resolution and also has narrower bandwidths in the red and near infrared that improve its sensitivity to chlorophyll and produces less distortion from atmospheric water vapor [25].

The Normalized Difference Vegetation Index (NDVI) measures vegetation cover, and over time can be used to monitor plant growth. NDVI is calculated as follows [26]:
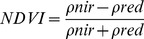
Where *ρ_nir_* is the reflectance in the near-infrared portion of the electromagnetic spectrum and *ρ_red_* is the reflectance in the red region. In the red region of the electromagnetic spectrum, sunlight is absorbed by chlorophyll, causing low red-light reflectance. In the near-infrared portion of the spectrum, the leaf's spongy mesophyll causes high reflectance. NDVI values range from −1.0 to 1.0, where healthy vegetation has increasingly positive values and decreasing negative values indicate rock, soil, snow, ice or clouds [27].

The Enhanced Vegetation Index (EVI) is a modification on NDVI that adds adjustment factors for soil and aerosol scattering. Liu and Huete [28] defined EVI as follows:
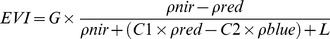
where *L* is a soil adjustment factor. *C_1_* and *C_2_* are coefficients that correct the red band for aerosol scattering by factoring in the blue band. Reflectance values for the near-infrared, red, and blue wavelengths are designated by *ρ_nir_*, *ρ_red_*, and *ρ_blue_*, respectively. Usually, *G* = 2.5, *C_1_* = 6.0, *C_2_* = 7.5, and *L* = 1.

Although MODIS Terra provides both NDVI and EVI products, we chose to analyze the EVI product for several important reasons. EVI is better at correcting for distortions caused by reflected light from particles in the air and from the ground cover below the vegetation. In areas with heavy vegetation, EVI is more sensitive to small changes and does not become saturated as easily as NDVI. NDVI responds mostly to variations in the red band which occur from chlorophyll, whereas EVI depends more upon the near-infrared band that is responsive to structural variations in the canopy [25].

Vegetation indices were not available for four studies prior to 1981. The only instrument acquiring daily global imagery was the AVHRR sensor on board the NOAA-6 satellite, which was launched in 1979. NOAA-6 has a daylight overpass time of 0730 hours whereas NOAA-7, launched in 1981, has a daylight overpass time of 1430 hours. NDVI products are not created from the NOAA satellites with early morning daylight overpass times (NOAA-6 and NOAA-8), because the lower solar zenith angle produces inconsistent and less intense radiance measurements than the afternoon solar illumination conditions from the other NOAA satellites [29]. Landsat multispectral scanner MSS offers an alternative to the AVHRR sensor for vegetation studies prior to 1981 [30]. However, due to its 18-day repeat cycle Landsat MSS does not produce enough cloud-free images to create a monthly time series for measuring seasonal vegetation dynamics [29].

Each study area was defined by locating the city on aerial imagery within Google Earth and creating a polygon around the city measuring 40 square kilometers that avoided large water bodies. ESRI's ArcMap 9.2 software and the Arc2Earth extension converted the polygon KML files to ArcGIS format. Bimonthly GIMMS data were acquired as geo-referenced TIF images in a geographic coordinate system, which are fully compatible with ArcGIS 9.2. Duke University's Marine Geospatial Ecology Toolset converted the monthly MODIS data from the HDF file format to ArcGIS rasters. For quality control, each study location polygon was overlaid onto the satellite imagery in conjunction with global country data to ensure proper alignment. We used Python scripts and ArcToolbox's Zonal Statistics function to create a time series of the mean vegetation index for each study area. For the GIMMS data, bimonthly statistics were averaged to create one monthly value.

### Data Standardization

The studies selected for this analysis used different measures for rotavirus outcome. Of the 39 studies, 25 studies (64.1%) presented outcome data as number of cases, 13 studies (33.3%) presented outcome as percent positive stools, and one study (2.6%) presented the outcome as incidence. In order to standardize different outcome measures the raw values were normalized into z-scores on a time series-by-time series basis. The z-score was calculated using the mean and standard deviation for the complete duration (all months) of each study as follows:
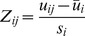
where *Z_ij_* is the z-score for the actual value for outcome, *u_ij_* (rotavirus cases, incidence or percent positive stools), for time series *i* in month *j* and 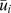 and *s_i_* are the mean and standard deviation for each time series, respectively. We also standardized exposure characteristics (temperature, precipitation and vegetation index) to adjust for location specific variations and assess relative associations [22].

### Data Analysis: Mixed Effects Models

The relationship between the normalized outcome and meteorological parameters was examined using a regression model adapted to time series data. We applied a linear mixed effects model to link the z-score of monthly rotavirus values with z-score of temperature, z-score of precipitation, and z-score of VI individually and control for the length of the time series at each study location. The mixed effects regression model was defined as follows:
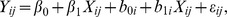
where *Y_ij_* is the rotavirus z-score, and *X_ij_* is the exposure of interest (temperature z-score, precipitation z-score, or VI z-score), the fixed effects: *β_0_* is the population intercept, *β_1_* is the population slope, and random effects: *b_0_* is the study intercept and *b_1_* is the study slope. The mixed effects model accounts for unequal lengths of times series included in the analysis, so studies which contribute less data have a smaller effect on the overall results. This model also allows us to explore the relationship between temporal fluctuations in rotavirus and meteorological conditions of each study as compared to the overall pattern for all studies. We examined the relationships overall for all locations as well as for tropical climates (Climate A) and humid mid-latitude climates (Climate C). As there were only two studies, for the same location, in the arid/semi arid (Climate B) category we did not examine relationships for this climate category.

We considered four regression models. Model 1 was defined as the synchronous model and assessed the association between rotavirus z-score and temperature, precipitation, and VI z-score individually. In Model 2, the exposure was lagged by one month to assess whether increased rotavirus rates are associated with temperature, precipitation and VI exposure from the previous month. Model 3 was the synchronous model (Model 1) adjusted for the distance from the equator. In Model 3, we included the square of the study location's latitude to emphasize proximity to the equator and the interaction with the exposure in the regression model as a fixed effect for each individual exposure predictors to control for the location's distance from the equator. By adjusting for the distance from the equator we can account, in part, for heterogeneity in the interaction between annual temperature and precipitation levels and overall climate characteristics. Finally, in Model 4 the exposure was lagged by one month and the model was adjusted for the distance from the equator. Regression parameters were tested at α = 0.05 significance level. All analysis was conducted using R Software (Version 2.9.2).

The regression parameters can be interpreted in terms of the z-score; a one unit change in z-score of an exposure variable is associated with an estimated change in z-score of dependant variable. A one unit change in z-score is equivalent to the standard deviation estimate of the given time series, thus to express the estimated effect in the original units of the explanatory variable the regression parameter can be divided by the value of the corresponding standard deviation estimate. Assuming that the majority of values of monthly measures of rotavirus infections are within the range of six standard deviations, we can then express the effect in terms of percent change by dividing the estimated effect in the original units of explanatory variable by 6 and multiplying the result by 100%.

### Data Analysis: Poisson Harmonic Regression

To evaluate varying seasonal patterns based on study location, we conducted a seasonality assessment. Annual seasonal patterns were examined for study locations that included rotavirus data for 3 or more years using Poisson harmonic regression. There were six study locations which included sufficient data for detailed seasonality analysis. Seasonality is characterized as systematic, periodic fluctuations within the course of a year. It is assessed by several parameters: 1) the time when the seasonal curve reaches its maximum, 2) annual maximum value (peak), and 3) annual minimum value (nadir) [31]. These seasonal parameters are calculated based on values predicted by the harmonic regression:
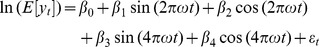
where *y_t_* is a time-series rotavirus z-score, *t* is time in months, *ω* is frequency (*ω* = 1/12), *β*_0_ is intercept, *β*_1_, *β*_2_, *β*_3_, and *β*_4_ are regression parameters, and *ε_t_* is the error term. We use two harmonics to account for the double peak seen in several time series. Using the estimate for the regression parameters, we calculated peak timing and intensity based on the δ – method [32]. The relative intensity, a measure of the shape of the seasonal pattern from peak to nadir, is calculated by dividing the estimated seasonal maximum value by the estimated seasonal minimum value.

## Results

**Figure 3:**
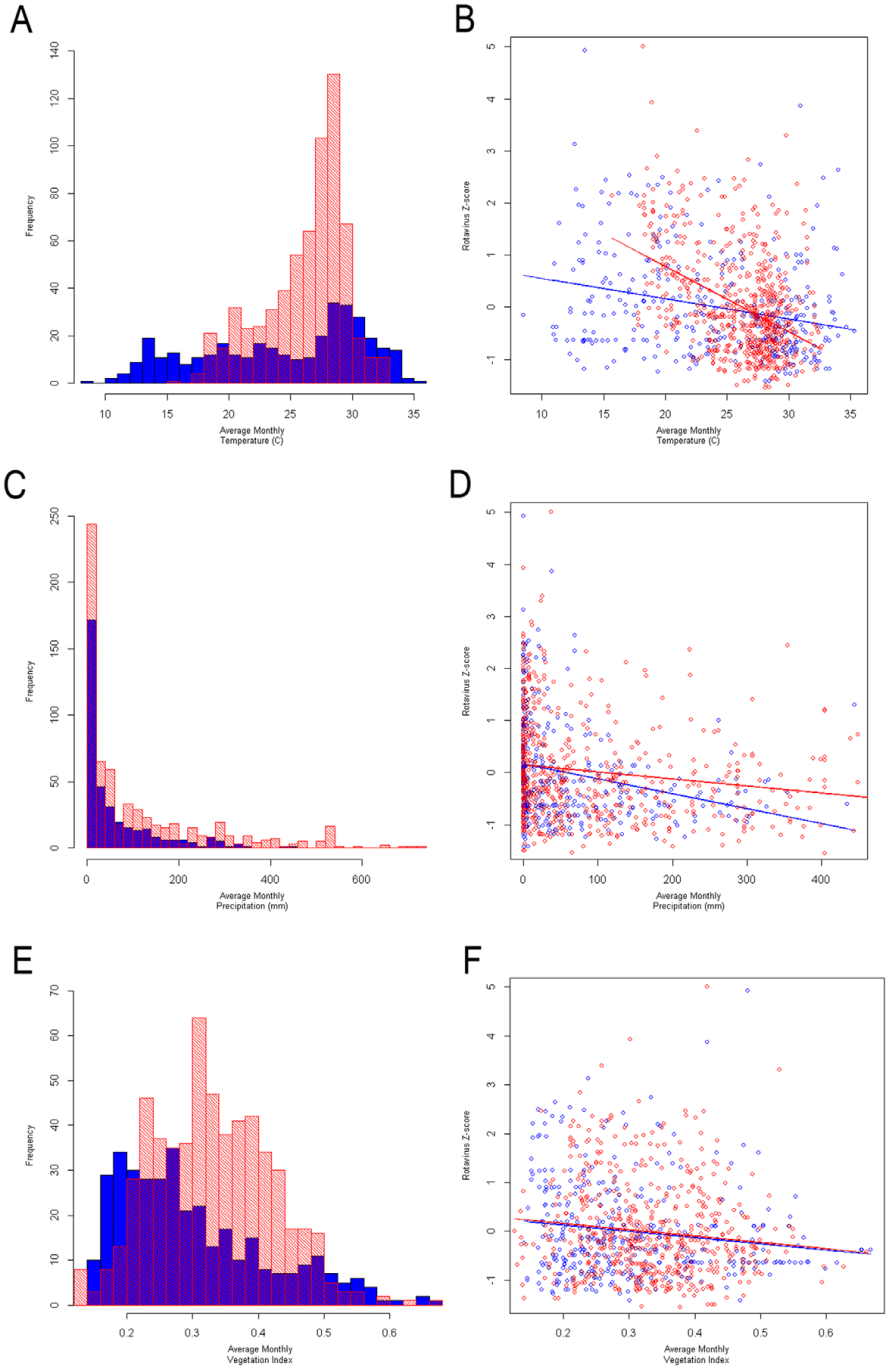
Histograms of independent variables and scatterplots showing relationships with rotavirus z-score. Histograms showing distribution of independent variables (temperature – Panel A, precipitation – Panel C, vegetation index – Panel E) by climate, tropical climates (A) in red and mid-latitude climates (C) in blue. Scatterplots showing the relationship between independent variables and rotavirus z-score (temperature – Panel B, precipitation – Panel D, vegetation index – Panel F) by climate, tropical climates (A) in red and mid-latitude climates (C) in blue.

A total of 47 time series were used in this analysis to assess the relationship between monthly rotavirus rate in South Asia with ambient temperature and precipitation. All study locations provided a total of 1046 months of data; on average, each study location had 22 months of data. The scatterplots of independent variables with rotavirus z-score demonstrate a stronger association in temperature for both climate categories than in precipitation and vegetation index (Figure 3). Descriptive analysis illustrates the average values of temperature, cumulative precipitation, and vegetation index for all study locations and also for each climate subcategory (Table 2). As all studies are from South Asia, there is little difference in the average temperature range by climate categories. The primary variation by climate category is seen in precipitation. The vegetation index is similar for moist tropical areas (A) and humid mid-latitude areas (C); but averaged much smaller values in the arid and semiarid region (B).

**Table 2:** Descriptive characteristics for all studies and by climate category.

Exposure Variable	All Studies	Climate A Moist tropical	Climate B Arid and Semiarid	Climate C Humid mid-latitude
Temperature (°C)	25.55±4.81	26.24±3.33	26.70±4.22	24.22±6.53
Precipitation (mm)	90.13±129.47	111.28±147.88	24.20±36.30	58.82±83.37
# of Time Series	47	30	2	15
Months	1046	649	36	361
Vegetation Index	0.31±0.11	0.33±0.90	0.13±0.02	0.30±0.11
# of Time Series	43	26	2	15
Months	972	575	36	361

Mean and standard deviation for temperature and precipitation, vegetation index, number of time series and number of months covered.

The highest levels of rotavirus are seen in the colder, drier months of the year (∼December–March). When assessing synchronized relationships (Model 1), all exposure parameters, temperature, precipitation, and VI z-score were significant negative predictors of rotavirus z-score and this relationship held for both the tropical (Climate A) and humid-mid latitude (Climate C) climates (Table 3). For all studies, a one unit increase in temperature z-score, precipitation z-score and VI z-score resulted in a −0.379 (95% CI: −0.435, −0.323), −0.246 (95% CI: −0.305, −0.188), and −0.253 (95% CI: −0.314, −0.193) unit decrease in the rotavirus z-score, respectively. Therefore, the overall effect of a 1°C decrease in monthly ambient temperature is associated with 0.078 unit increase above the annual rotavirus level, which is proportional to 1.3% increase in rotavirus infections. Similarly, a decrease of 10 mm in precipitation is associated with 0.3% increase in rotavirus infections. A decrease of 0.1 units in vegetation index for a seasonal change – compared to the annual norm of 0.3 – is associated with a 3.8% increase above the annual rotavirus level. In the tropical climates (Climate A) temperature is more strongly associated with a decrease in rotavirus and in the humid mid-latitude climate (Climate C) temperature and precipitation have similar negative association with rotavirus z-score. In the tropical climates a decrease of 1°C from annual average of 26.2°C in monthly ambient temperature is associated with 0.13 unit increase above the rotavirus annual level, or a 2.2% increase in rotavirus infections, while in the humid mid-latitude climate (Climate C) a decrease of 1°C in temperature below annual mean of 24.2°C results in lesser but significant increase of 0.7% increase. VI demonstrated a similar negative association with rotavirus z-score in both climates.

**Table 3:** Regression parameters and confidence intervals for Models 1–4 for all studies and by climate category (analysis was not conducted for Climate B because there was only one location in this category).

	Exposure Variable	All Studies		Climate A Moist tropical		Climate C Humid mid-latitude	
		Estimate	95% CI	Estimate	95% CI	Estimate	95% CI
**Model 1** †	Temperature	−0.379*	(−0.435, −0.323)	−0.444*	(−0.513, −0.375)	−0.310*	(−0.408, −0.211)
	Precipitation	−0.246*	(−0.305, −0.188)	−0.259*	(−0.334, −0.185)	−0.271*	(−0.370, −0.171)
	Vegetation Index	−0.253*	(−0.314, −0.193)	−0.240*	(−0.320, −0.161)	−0.281*	(−0.380, −0.182)
**Model 2** ‡	Temperature	−0.288*	(−0.347, −0.229)	−0.363*	(−0.434, −0.291)	−0.209*	(−0.314, −0.105)
	Precipitation	−0.186*	(−0.242, −0.130)	−0.209*	(−0.284, −0.134)	−0.173*	(−0.261, −0.084)
	Vegetation Index	−0.154*	(−0.217, −0.091)	−0.161*	(−0.242, −0.081)	−0.135*	(−0.241, −0.029)
**Model 3** §	Temperature	−0.331*	(−0.552, −0.111)	0.173	(−0.094, 0.441)	−2.051*	(−3.138, −0.963)
	Precipitation	−0.190	(−0.421, 0.041)	−0.085	(−0.378, 0.208)	−0.759	(−1.874, 0.356)
	Vegetation Index	−0.076	(−0.319, 0.167)	0.117	(−0.198, 0.431)	−1.255*	(−2.363, −0.147)
**Model 4** ¶	Temperature	−0.328*	(−0.558, −0.098)	0.128	(−0.151, 0.407)	−1.858*	(−3.022, −0.695)
	Precipitation	−0.040	(−0.247, 0.194)	0.014	(−0.282, 0.310)	0.545	(−0.182, 1.273)
	Vegetation Index	−0.076	(−0.319, 0.167)	0.302	(−0.015, 0.619)	−0.955	(−2.122, 0.212)

Significant parameters (p<0.05) are indicated with an asterisk (*).

† Synchronous model.

‡ Model with exposure lagged by one month.

§ Synchronous model adjusted for distance from the equator.

¶ Model with exposure lagged by one month and adjusted for distance from the equator.

All three exposures remained significant when lagged associations were considered (Model 2) though the strength of the relationships decreased. This was also seen for both climate categories (Table 3). Overall for all locations, when adjusting for latitude (distance of the study location from the equator (Model 3)) only the relationship with temperature z-score remained significant suggesting that the association is stronger for locations farther from the equator. This is seen clearly when considering the relationship by climate category; in the humid mid-latitude climate (Climate C), which is further from the equator, the relationship with temperature is stronger than in the tropical climate (Climate A), which are locations closer to the equator. Overall for precipitation z-score and VI z-score the relationship becomes insignificant after adjusting for latitude. Similar associations were seen after adjusting for latitude and lagging the exposure variable (Model 4).

**Figure 4:**
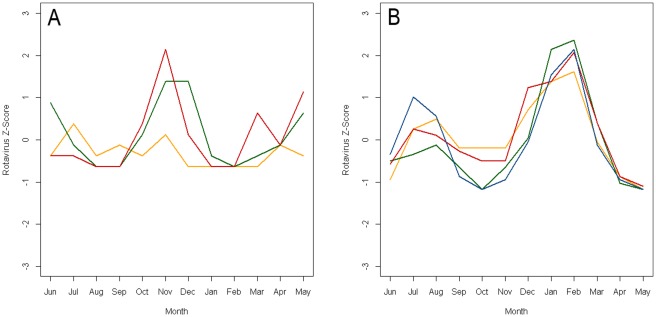
Annual time series of rotavirus z-score superimposed for years of the study for two locations. Panel A is data for a study conducted in Chandigarh, India [44] and Panel B is data for a study conducted in Matlab, Bangladesh [65]. Data are shifted by 6 months in order to center the winter peaks.

Poisson harmonic regression was used to analyze the seasonal patterns for six time series which provided monthly rotavirus data for more than 3 years. The seasonal patterns vary by location but typically demonstrate a peak in the winter months. The seasonality assessment demonstrated a winter peak for all the locations (Table 4). The estimate for peak timing of seasonal peak ranged from late October to late February. Figure 4 shows the annual time series of rotavirus z-score superimposed for the years of the study for 2 locations, Chandigarh, India which is in the humid mid-latitude climate (C) and Matlab, Bangladesh which is in the tropical climate (A).

**Table 4:** Estimated peak timing from Poisson harmonic regression for studies with more than 3 years of data including study reference, study location, study duration, estimated peak and 95% confidence interval, p-value, calendar interpretation of estimated peak and relative intensity for both estimated peaks.

			Estimated Peak 1				Estimated Peak 2			
Study Reference	Location (City, Country)	Study Duration (months)	Estimated Peak (95% CI)	P-Value	Peak Estimate by Calendar	Relative Intensity	Estimated Peak (95% CI)	P-Value	Peak Estimate by Calendar	Relative Intensity
[44]	Chandigarh, India	46	12.99 (11.42, 2.56)	0.326	4^th^ week of December	1.12	6.31 (5.84, 6.78)	0.361	2^nd^ week of June	1.48
[50]	New Delhi, India	84	11.25 (10.06, 12.44)	0.291	1^st^ week of November	1.21	8.20 (7.72, 8.54)	0.380	1^st^ week of August	1.47
[63]	Dhaka, Bangladesh	48	11.84 (11.65, 12.04)	0.044	4^th^ week of November	1.61	8.11 (7.69, 8.54)	0.351	1^st^ week of August	1.41
[65]	Dhaka, Bangladesh	65	12.93 (12.70, 1.16)	0.0003	4^th^ week of December	2.00	7.95 (7.82, 8.08)	0.006	4^th^ week of July	1.57
[65]	Matlab, Bangladesh	65	2.76 (2.52, 3.00)	0.001	4^th^ week of February	1.95	8.29 (8.19, 8.39)	0.009	2^nd^ week of August	1.52
[73]	Pune, India	48	1.05 (12.71, 1.38)	0.059	1^st^ week of January	1.56	11.51 (11.31, 11.71)	0.374	3^rd^ week of November	1.40

## Discussion

Our findings provide a quantitative link between the rate of rotavirus and meteorological parameters in South Asia. We found that higher rate of rotavirus is associated with cold, dry months. Our study demonstrates that the strength of these associations vary by climate category. The use of standardized z-scores allows for direct comparisons of detected effects across geographical zones with different weather conditions and across various measures of disease. This study also demonstrates the utility of the remote sensing vegetation index in assessing seasonal associations.

The seasonal pattern seen in rotavirus varies by climatic region and is also associated with local weather. Our findings agree with a review of studies in tropical regions which found that a reduction of rotavirus rates was associated with increases in temperature and precipitation [8]. Our analysis focuses on South Asia, which includes both temperate and tropical areas, whereas the Levy et al. study includes just the tropics; only seven out of 39 studies were included in both analyses. The similarity of findings indicates that both studies capture a unique and stable phenomenon suggesting an effect of climate and local weather on the seasonal pattern of rotavirus infection: an increase in rate of rotavirus in cooler and drier times of the year for a given location. The Levy et al. study acknowledged the diversity of climates prevalent even within the defined tropical band [8]. In our study we controlled for various climatic regions by classifying each study using Köppen Climate Classification. Most of the study locations we examined fell into only two broad classifications, tropical climates (Climate A) and humid mid-latitude climates (Climate C). With the limited number of studies we were only able to use the broadest categories though the Köppen Climate Classification system provides more detail. Within the broad tropical climate (A) category there are three subcategories which are distinguished by precipitation patterns and in the mid-latitude climate (C) category there are six subcategories which are distinguished by type of summer as well as precipitation patterns [23]. However, we did demonstrate different degree of association between meteorological characteristics and rotavirus prevalence by climate category. In tropical climates (Climate A) an increase in temperature was associated with a greater decrease in rotavirus than in humid mid-latitude climates (Climate C). We also found that temperature is a stronger predictor of rate of rotavirus after controlling for the study latitude or distance from the equator in the humid mid-latitude climate (C) whereas the relationship decreased for the moist tropical climate (A); this suggests the viral transmission pattern might depend on broad range of environmental conditions, which can also be drivers for temporality in social behaviors and health practices.

The primary reasons for seasonal fluctuation in rotavirus prevalence are still unknown. Typically for a virus, it is not expected that the seasonal relationship would be primarily driven by a favorable environment for the pathogen. Temporal and geographic trends have been better recognized in temperate than tropical climates, perhaps because though temperatures vary across seasons, temperatures do not reach the lower levels seen in temperate climates, or patterns of disease prevalence are not as marked. It has been suggested the seasonal pattern seen in rotavirus may be driven by airborne transmission of the disease [16], [33], [34]. The drop in humidity and rainfall dries the soils which may increase the aerial transport of the contaminated fecal matter. In the U.S. the seasonal pattern in rotavirus shifts by geographic location with peak activity occurring first in the Southwest from October through December and last in the Northeast between March and May [13], [34]. Influenza has also demonstrated strong seasonal patterns [15], [35], [36] and associations with humidity [37]. A seasonal pattern similar to that of rotavirus has been demonstrated for influenza in the U.S., with peak activity beginning in the Southwest in mid-December and shifting to the Northeast towards mid-January [36].

The seasonal pattern seen in rotavirus may be influenced by socio-demographic factors. Pitzer et al. suggest that the seasonal pattern seen in rotavirus in the U.S. is influenced by spatiotemporal patterns in birth rate and that young children experiencing their first infection of rotavirus are the primary drivers of epidemics and therefore of the seasonal patterns [9]. As mentioned by Bobak et al., in populations with reliable data, birth rates vary by season of the year [38]. In the U.S. most births occur in the summer and early fall and the fewest births are seen in the spring which can be explained by temperature and light effects [12], [39]. Seasonal patterns in birth rates have been demonstrated in South Asia, though specific regional patterns are not clear [12], [39]. In regions with distinct periods of very high temperatures, a drop in births at this time can be noticeable, especially in developing countries, which may, in part, explain our findings in the tropical (A) climates. Seasonality of newborn susceptibility might be insufficient to warrant disease seasonality.

It is likely that seasonal patterns can be reinforced by changes in rotavirus genotype. In the temperate climate of England and Wales, where rotavirus infections are highly seasonal, a child's risk of a laboratory-confirmed rotavirus infection in the first year of life was significantly higher for children born in summer compared to those born in the winter [40]. The authors suggested that maternal immunity and age-specific levels of exposure to rotavirus could explain the findings. Our preliminary findings suggest that the seasonal pattern for rotavirus may vary by strain type [41]. A similar meta-analysis approach may be used to assess the differing seasonality by strain type.

School schedules and holiday travel may also affect the seasonal pattern of rotavirus infections by changing the probability of exposure to the virus. Other viral diseases, influenza in particular, have demonstrated associations with holiday travel and school schedules [42]. In many countries, vacations and family travels are somewhat synchronized and it may be difficult to separate the independent effects. Given the potential airborne transmission of rotavirus it can be expected that the seasonal pattern may be driven by crowdedness. Rotavirus is most prevalent in children under 5 years of age. Over 70% of the studies considered were conducted in children less than 5 years of age and therefore, our results are mostly driven by the seasonal patterns seen for this age group. The degree to which seasonal patterns in children under 5 years of age in South Asia can be affected by school schedules and holiday travel needs to be further investigated.

The studies used in our analysis covered a large time span (1976–2009) and it was difficult to gather temperature and precipitation data specific to the study time for the earlier study periods and studies in more rural areas. For the studies in which averages were used, temperature and precipitation may not represent the weather specific to the year for which rotavirus data is available. This may limit the precision of our estimates of associations with meteorological factors. Humidity has also been shown to be associated with rotavirus seasonality [8], [11]. An analysis of birth cohort data from Vellore, India, demonstrated a positive correlation between humidity and the strain-specific incidence of rotavirus diarrhea [41]. However, humidity is another factor for which consistent data was difficult to obtain for all study locations. To overcome this limitation, we consider the use of remote sensing data to provide a proxy for meteorological characteristics for areas in which direct measures of temperature and precipitation are not available. The Vegetation Index (VI) is a measure of the combined effect of temperature and precipitation and is therefore a strong proxy for humidity as well. Study specific temperature and precipitation data was available for only 75% of study months whereas VI measures were available for 93% of study months. A strength of this analysis is the demonstration of the use of VI as a proxy for the combined effect of temperature and precipitation.

The precision of our estimates may also be affected by the coarse monthly temporal aggregation. The incubation period for rotavirus is short, approximately 2 days, and the disease is highly infectious which suggests that the seasonal peak of disease may be very short [33]. A finer temporal aggregation, of weeks or days, for rotavirus data would allow us to capture the short peak more accurately using Poisson harmonic regression. Our sub-analysis of seasonality characteristics in locations with sufficiently long time series data demonstrates that the form of the seasonal pattern might be more complex then typically observed in temperate climates, which usually exhibit one well defined winter peak. The ongoing global rotavirus monitoring and surveillance efforts will soon provide valuable information for detailed seasonality characterization.

The seasonal pattern seen in rotavirus may be influence by our ability to measure infection in a systematic manner. Due to the long time span covered by studies used in this analysis, 1976–2009, various diagnostic methods were used for rotavirus. Enzyme immunoassay is currently the most common technique while earlier studies were likely to use electron microscopy. It is estimated that a child with rotavirus diarrhea excretes 10^9^ to 10^12^ viruses per gram of stool during the acute phase of diarrhea [16], [33]. The enzyme immunoassay, which is based on VP6 antigen detection, and electron microscopy, by which the 70 nm viral particles are visualized, both have a limit of detection of 10^5^ to 10^6^ viral particles per gram of stool, and hence are unlikely to miss cases of rotavirus diarrhea in acute illness. Since both methods have similar limits of detection, it is unlikely that differences in methods would affect detection of seasonal patterns of infection. The use of various diagnostic methods required over time required standardization of data to z-score. While necessary for this analysis, this approach reduces the effect of temporal heterogeneity but can't eliminate potential bias, which might lessen the observed effects. To improve our understanding of rotavirus seasonality, better capacity and standardization of global monitoring and surveillance efforts are required [17].

Understanding seasonal patterns of rotavirus allows for testing of efficacy of vaccines currently in use [5], [6]. The details of seasonal patterns can be used to target vaccination programs, test efficacy of the vaccine implementation programs and the vaccine itself [6]. In particular, it may be useful to consider appropriately timed immunization booster programs in settings which have reported poor efficacy of rotavirus vaccine which wanes further in the second year of life, and have demonstrated strong seasonality [43]. The typical schedule for rotavirus immunization in developing countries includes vaccination at 6, 10, and 14 weeks of age and is constrained by the very young mean age of primary rotavirus infections and the increased background risk of adverse events after six months of age. However, if booster vaccination programs were to be considered for older children lacking immunity, pre-rotavirus season vaccination may be substantially more effective than post-season vaccination. Proper assessment of seasonality and dependency on climate characteristics also provides insights on effects of vaccination strategies, including potential shifts in seasonal peaks and duration of outbreaks.

### Conclusion

In this study, we found the highest rate of rotavirus was seen in the colder, drier months in South Asia. This study capitalizes on previously published methodology for assessing the relationships between meteorological characteristics and disease outcomes [22], [31]. With expansion of remote sensing and improved weather monitoring, global weather forecasting on a very refined spatial scale is feasible. A better understanding of the links between disease ecology and environmental conditions warrants the discovery of underlying governing principles of transmission of rotaviral infection. Based on the findings, higher of rates of rotaviral infection during the relatively cold and dry periods in South Asia, innovative strategies for global disease forecasting, early warning systems, timing of vaccination programs, and other preventive measures can be developed at the regional level.
